# Supplementary material for: Chromothripsis during telomere crisis is independent of NHEJ, and consistent with a replicative origin
Source: Genome Res. 2019 May;29(5):737–49. doi: 10.1101/gr.240705.118 (PMC6499312; doi:10.1101/gr.240705.118)
Supplement: Supplemental Material [file supp_gr.240705.118_Supplemental_file_1.zip › contigs/annotated_contigs/DB73/contig.3.DB73_length_440_mean_cov_6.69545454545.docx]

**DB73_length_440_mean_cov_6.69545454545**

```
AGAAAAGACCCACTAAAGATCAAAAATTGGTGAGTATCTATAATAGACTTTTGTTCATTACTTAACCTTTTCTTTTACATTGACCCCCT
 >chr2:222981429-222981584 - E=1e-78 p=0e+00
CATCACCATTTGGGAAAAATCTTTCCCTATGGAGATATAG|ATATCTATATCTATATTTATATCTAT|CTATCTATCTATCTATCTATC
                                         >chr2:222981161-222981419 - E=6e-124 p=0e+00
TATCTATCTATCTATCATCTATCTATCTATCTGTATCCTTGTTTTTTGTGTGTTTGTTTTTTCAATTGTCTAAGTTGGGTCTTCTGGCC

TTTTGATGATTTTGCATCTTTGATTTCCTTCCTTCCTTCCTTCCTTCCTTCCTTCCTTCCTTTCTTTTCTTTTCTTTTCTTTCTTTCTT

TCTTTCTTTCTTTCTTTCTTTCTTTCTC|TTTCTTT|TTCTTTCTTTTTTTCTTTCTTTTTTTTTTTCTTTTTTTTTTTTTTTTTTTT                             >chr10:89379325-89379383 - E=2e-17
```
